# Correction to “Features and Prognosis of Patients With Retroperitoneal Fibrosis Developing Fibrosing Mediastinitis: Case‐Control Study and Systematic Review”

**DOI:** 10.1002/acr2.70149

**Published:** 2025-10-26

Ding, Y. , Li, Z. , Liu, S. , Li, M. , Ren, Y. , Xu, K. F. , Luo, C. , Pan, C. , & Gao, H. Features and Prognosis of Patients With Retroperitoneal Fibrosis Developing Fibrosing Mediastinitis: Case‐Control Study and Systematic Review. ACR open rheumatology, 2025；7(8), e70065. 10.1002/acr2.70065 40728059 PMC12305350

Due to changes in the authorship, in the footnote on the first page, the text “Drs Ding and Li contributed equally to this work.” was incorrect. This should have read: “Drs Ding, Li, and Liu contributed equally to this work.”

In paragraph 37 of the “RESULTS” section, the text “and mechanistic target of rapamyci” was incorrect. This should have read: “and mechanistic target of rapamycin”. In paragraph 42 of the “RESULTS” section, the text “Clinical features, management, and prognosis of patients with RP9F and MF.” was incorrect. This should have read: “Clinical features, management, and prognosis of patients with RPF and MF.”. The online version of this article has been corrected accordingly.

We apologize for this error.

